# Personality characteristics associated with satisfaction with healthcare and the wish to complain

**DOI:** 10.1186/s12913-022-08688-7

**Published:** 2022-11-01

**Authors:** Søren Birkeland, Marie Bismark, Michael J. Barry, Sören Möller

**Affiliations:** 1 Department of Clinical Research, University of Southern Denmark and Open Patient Data Explorative Network, Odense University Hospital, J. B. Winsløws Vej 9 a, 3. Floor, 5000 Odense C, Denmark; 2 Melbourne School of Population and Global Health, University of Melbourne, Melbourne, Australia; 3 Division of General Internal Medicine, Massachusetts, General Hospital & Harvard Medical School, Boston, USA

**Keywords:** Communication, Health law, Litigation, Malpractice Complaints, Personality, Satisfaction

## Abstract

**Background:**

There is increasing evidence that satisfaction with healthcare and complaint rates vary with patients’ socio-demographic characteristics. Likewise, patient personality might influence the perception of health care; however, empirical research has been scarce. The aim of this study was to investigate associations between health care user personality and satisfaction with care and urge to complain.

**Methods:**

This study is a randomized survey among Danish men aged 45 to 70 years (*N* = 6,756; 30% response rate) with hypothetical vignettes illustrating different courses of healthcare. Assuming they received the care described in vignettes, participants rated their satisfaction and wish to complain on a five-point Likert scale. Information on personality characteristics was obtained through self-reports using the standardized Big Five Inventory-10 (BFI-10).

**Results:**

In multivariate analyses, we found respondents with higher scores on the agreeableness dimension expressing greater satisfaction with care (Likert difference 0.06, 95% CI 0.04 to 0.07; *p* < 0.001) and decreased wish to complain (-0.07, 95% CI -0.08 to -0.05; *p* < 0.001) while high neuroticism scores were associated with less satisfaction (-0.02, 95% CI -0.03 to -0.00, *p* = 0.012) and an increased wish to complain about healthcare (0.04, 95% CI 0.03 to 0.06, *p* < 0.001). Interaction analyses could demonstrate no statistically significant interaction between the level of patient involvement in decision making in the scenarios and the effect of personality on respondents' satisfaction and wish to complain. Generally, however, when adjusting for personality, respondents’ satisfaction increased (*P* < 0.001) with greater patient involvement illustrated in case scenarios while the wish to complain decreased (*P* < 0.001).

**Conclusion:**

Our findings suggest low agreeableness and high neuroticism scores are associated with lower patient satisfaction with healthcare and increased wish to complain. Irrespective of personality, however, the wish to complain seems responsive to changes in patient involvement, underscoring the importance of inclusive healthcare communication.

**Supplementary Information:**

The online version contains supplementary material available at 10.1186/s12913-022-08688-7.

## Background

Most health care professionals strive to do their best in providing their patients with the medical care they need. From time to time, however, things go wrong. A patient's satisfaction with healthcare, according to some models, is a function of expectations and perceived outcomes [1]. For example, patients sometimes receive the wrong diagnosis or treatment, the right treatment in a wrong way, or feel that they were not properly informed about their options or the risks of treatment. When dissatisfaction is triggered, various ‘complaint behaviors’ may occur (like voicing of one’s dissatisfaction, private responses in terms of, e.g., word-of-mouth communication to friends and relatives, and third party responses like filing a malpractice suit [2]). Whatever the mechanism may be, patients are sometimes dissatisfied with their healthcare and a portion of these patients choose to pursue legal action, be it through, e.g., state boards handling disciplinary cases or through legal remedies with the opportunity for receiving tort compensation (together referred to as “complaints”) [3, 4].

Research has shown that the rates of patient complaints are higher among certain specialties and health professional demographics, with older male surgeons featuring highly in many complaint datasets [5, 6]. Likewise, there is growing evidence that some socio-demographic characteristics of patients are associated with a higher risk of complaints [4, 6–11]. Anecdotally, when talking about dissatisfaction with healthcare, practitioners sometimes point to their experience with ‘difficult patients’ although this notion might deserve renaming to ‘difficult clinician-patient relationships’ [12]. A few studies have suggested satisfaction with healthcare may be associated with patients’ personality type [13–15]. Similarly, it has been put forward that personality might predict the proclivity to complain about care [16, 17]. However, virtually no empirical evidence exists to support this association. Similarly, no previous research has assessed to what extent the effect of personality on patients’ health care experience varies with improved communication and the degree of patient involvement. In this study, we aimed to fill this knowledge gap. In a large national survey using hypothetical case vignettes and a standardized measure of personality, we studied health care users’ satisfaction with care and urge to complain. We hypothesized that satisfaction with healthcare and the wish to complain may be associated with personality factors and can be modulated through greater involvement in healthcare decision-making.

## Methods

**Fig. 1:**
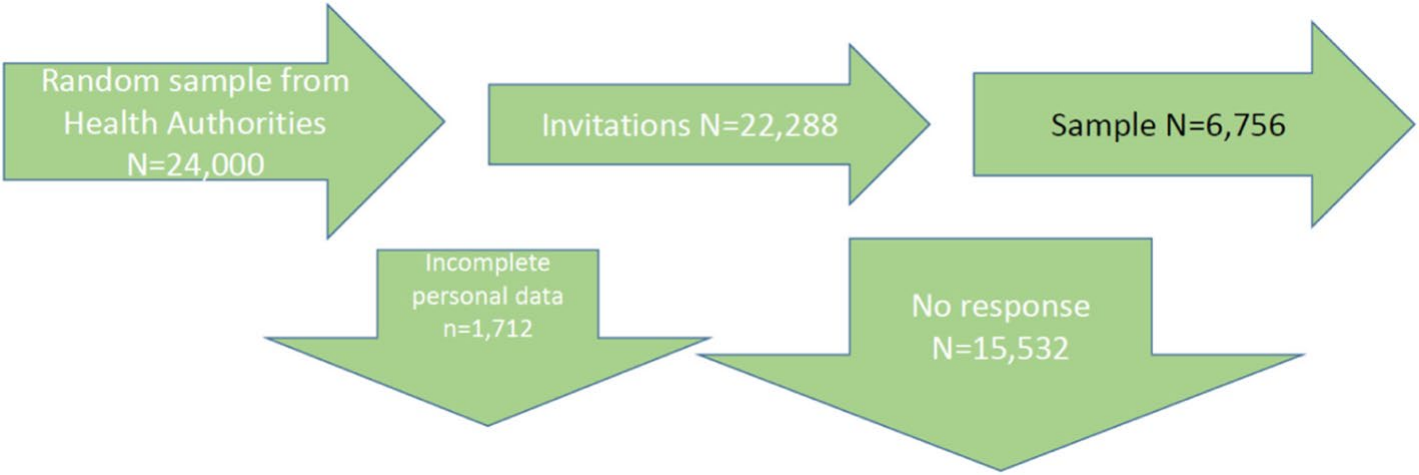
Inclusion of survey participants

This paper describes part of a larger project that examines predictors for healthcare satisfaction and complaint behavior. Following public and patient involvement in the design, we developed a cross-sectional survey [11]. A random sample of 24,000 men aged 45 to 70 years was selected from the national register held by Danish health authorities (please see flowchart in Fig. 1; for sample size considerations, please see [18]).

We sent invitations in two waves and ultimately, 6,756 people completed the survey (response rate = 30 percent). Comparisons to national and international datasets regarding baseline socio-demographics, control preference, and personality characteristics, showed reasonable representativeness of our sample [19, 20]. We used Research Electronic Data Capture (REDCap) for the survey and distributed it through a digital mail box used by authorities for secure communication with citizens [21]. As a model clinical ‘intervention’, we used hypothetical case vignettes concerning prostate-specific antigen (PSA) screening for prostate cancer in men. We randomized respondents into one of 30 distinctive scenarios.

The core of all scenarios was identical but they varied regarding the patient-doctor communication described with different levels of patient involvement in the decision about having a PSA test (5 levels from no information at all, nudging against intervention, neutral information provision, nudging in favor of intervention, and involvement using Shared Decision Making, SDM, and a decision aid), the choice to have the intervention or not, and outcomes (no cancer, diagnosis of a treatable cancer, and diagnosis of an eventually lethal cancer). Vignettes are described in further detail elsewhere [18, 22] (please also see supplementary information). Participants indicated their inclination to initiate a complaint about healthcare if subject to the scenario illustrated using a 5 point Likert scale ranging from 1 (very unlikely) to 5 (very likely). In Denmark, patients may complain about health care by initiating a malpractice suit (through a ‘patient injury compensation organization’) or by complaining to a state medical (‘disciplinary’) board without claiming compensation, or may choose to do both. Therefore, we included the following items: *'’How likely is it that you would claim compensation?*’ and *'How likely is it that you would complain about the doctor's care?*' In our analyses of data, we decided not to investigate the two ratings separately, as the distinction between compensation claims and complaints may vary among countries. Hence, a simple average of ratings was used to provide an overall estimate of the health care user’s wish to complain.

Participants also responded to questions regarding socio-demographic characteristics and personality. We used the ten item Big Five Inventory (BFI-10) to measure personality. This measure uses short phrases to assess the most prototypical traits associated with each of the Big Five dimensions [23, 24]. Explained in brief, the five factor model personality dimensions encompass different traits. ‘Extraversion’ describes individuals who are assertive, energetic, and talkative. ‘Agreeableness’ implies personality styles with affection, sympathy, and kindness. ‘Conscientiousness’ is characteristic of individuals who are organized and thorough. Individuals with high ‘Neuroticism’ scores often are moody, tense, and anxious and ‘Openness’ characteristics describe individuals who are imaginative and insightful and have many interests. Individuals express varying levels of each of the traits. While the interplay among traits is more difficult to assess, one trait often dominates. Respondents rated each BFI item on a 5-point scale ranging between strong agreement and strong disagreement. BFI scores were calculated as the respondent’s average item response for items related to each of the five dimensions.

We investigated the association between BFI scales and outcome measures (satisfaction and wish to complain) by linear regression. We carried out *i*) models including each BFI scale separately and only adjusted for scenario, handling the 30 vignettes as 30 different groups, hence including communication style, decision and clinical outcome (“crude univariate”), *ii*) models adjusting for respondent’s education, work status and presence of chronic illness (“adjusted univariate”), and *iii*) an adjusted model furthermore including all five BFI scales in the same model (“multivariate”). Moreover, we carried out a linear regression including interaction between BFI scales and level of patient involvement in decision-making (still adjusting for final decision and clinical outcome), to determine if communication modified the association between personality traits and outcomes. Due to non-normality of residuals we estimated confidence intervals and *p*-values by bootstrapping with 1000 iterations. A multiple testing correction was applied by the Bonferroni-Holm method separately for univariate and multivariate analyses.

## Results

The average age of respondents was 59.1 years (SD 7.3 years). Seventy-nine percent of respondents were married and two-third of respondents (66%) either had vocational, short-term higher education (< 3 years), or a bachelor level or higher education. Similarly, 66% were in a current job. Participants generally were satisfied with the healthcare described in vignettes (average Likert rating 3.92; 95% CI 3.90; 3.94) and ‘unlikely’ to complain (average Likert rating 2.08; 95% CI 2.06; 2.10)**.** Associations between BFI dimensional scores and satisfaction and complaint likelihood are shown in Table 1. For instance, a one point higher ‘agreeableness’ score was associated with a 0.07 decrease on the complaint likelihood score (for descriptive information on the scales used, see Table 2). Hence, a maximum ‘agreeableness’ score increase from 2 to 10 would be associated with roughly a half point decrease in complaint likelihood (e.g. from ‘even chance’ of a complaint to complaint ‘unlikely’). In univariate analyses controlling for respondents’ education, work status, chronic illness, the healthcare scenario, extraversion, agreeableness, and conscientiousness, all were significantly associated with higher satisfaction and lower wish to complain, while the opposite was the case with neuroticism. In the adjusted multivariate analyses including all BFI scales, we found higher agreeableness to be associated with more satisfaction and decreased wish to complain. On the contrary, higher BFI neuroticism scores were associated with less satisfaction and an increased wish to complain about healthcare.

**Table 1:** Associations between BFI dimensional scores and satisfaction and complaint likelihood, adjusted analyses ^a, b^

**Outcome**	**BFI dimension score**	**Adjusted univariate**		**Multivariate**^e^	
		**Coef. (95% CI)**	***P*-value**	**Coef. (95% CI)**	***P*-value**
Satisfaction with health care described in vignette	Extraversion	0.02 (0.01; 0.03)	0.004^d^	0.00 (-0.01; 0.01)	0.857
	Agreeableness	0.06 (0.05; 0.08)	< 0.001^d^	0.06 (0.04; 0.07)	< 0.001^d^
	Conscientiousness	0.02 (0.01; 0.04)	0.001^d^	0.01 (-0.00; 0.03)	0.121
	Neuroticism	-0.03 (-0.04; -0.02)	< 0.001^d^	-0.02 (-0.03; -0.00)	0.012
	Openness	-0.00 (-0.02; 0.01)	0.419	-0.00 (-0.01; 0.01)	0.721
Likelihood of complaint^c^	Extraversion	-0.01 (-0.02; 0.00)	0.130	0.01 (-0.00; 0.03)	0.051
	Agreeableness	-0.07 (-0.09; -0.06)	< 0.001^d^	-0.07 (-0.08; -0.05)	< 0.001^d^
	Conscientiousness	-0.02 (-0.04; -0.01)	0.001^d^	-0.01 (-0.03; 0.00)	0.093
	Neuroticism	0.04 (0.03; 0.05)	< 0.001^d^	0.04 (0.03; 0.06)	< 0.001^d^
	Openness	0.01 (-0.00; 0.02)	0.241	-0.01 (-0.02; 0.00)	0.096

^a^Adjusted for respondent’s education, work status, chronic illness and for scenario

^b^Crude unadjusted analyses can be found in supplementary information

^c^Combined likelihood of compensation claim and disciplinary complaint

^d^Significant on 0.05 level after correction for multiple testing

^e^Adjustments including all BFI dimensions

**Table 2:** Descriptive information on the scales used in study

	Observations	Range	Mean (SD)	Crohnbach’s alpha
Outcomes				
Satisfaction with health care described in vignette	6,755	1 to 5	3.92 (0.82)	NA (Single question)
Likelihood of complaint	6,755	1 to 5	2.08 (0.87)	0.86
Explanatory variables				
Extraversion	6,756	2 to 10	7.29 (1.68)	0.62
Agreeableness	6,756	2 to 10	7.32 (1.22)	0.24
Conscientiousness	6,756	2 to 10	7.82 (1.33)	0.42
Neuroticism	6,756	2 to 10	4.68 (1.59)	0.63
Openness	6,756	2 to 10	5.87 (1.65)	0.14

**Fig. 2:**
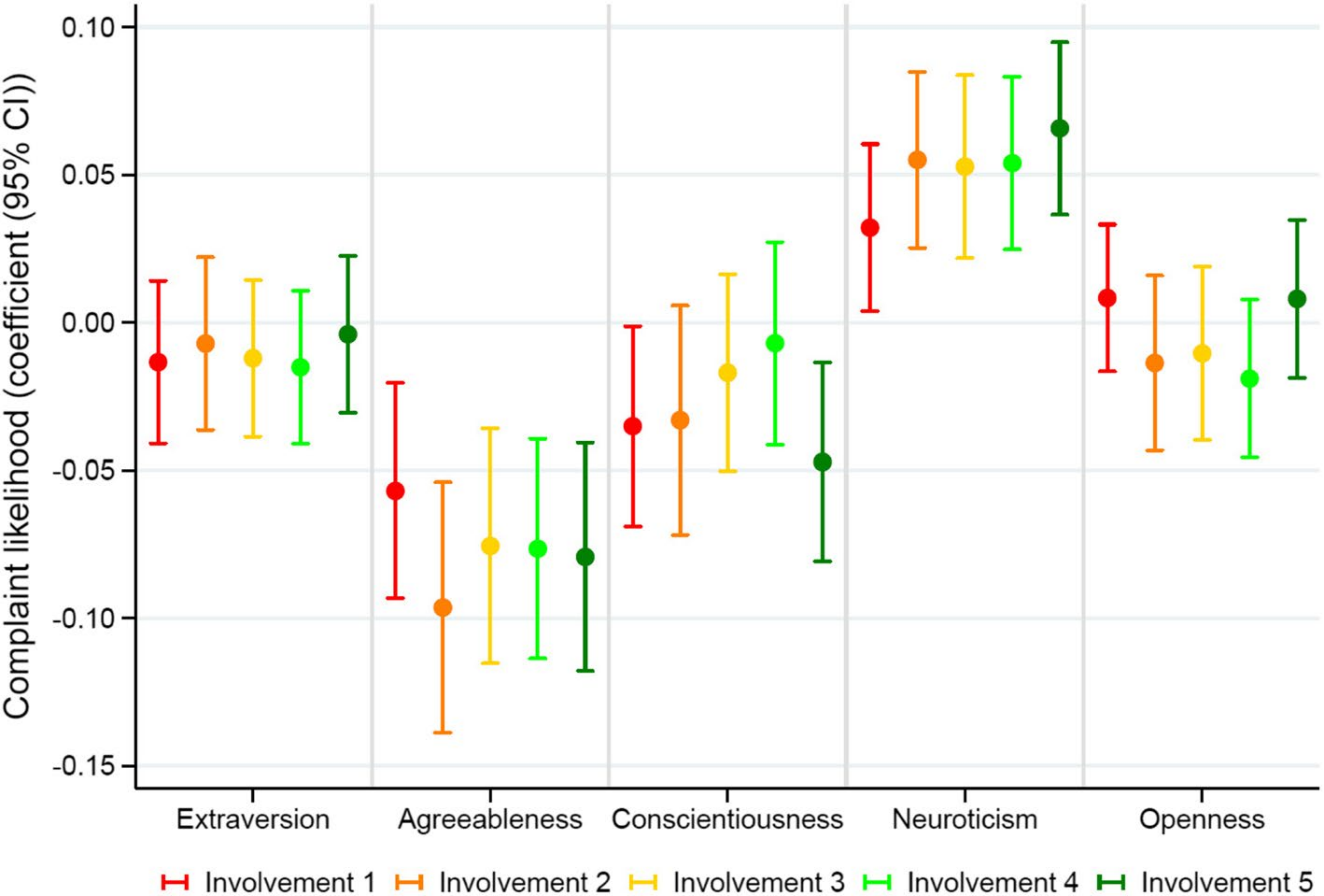
Interactions among personality, level of involvement, and the wish to complain. Involvement 1: No information, “- 2 “ information with nudging against intervention, “-3” neutral information, “-4”, nudging in favor of intervention, and “-5” involvement using Shared Decision Making and a decision aid

In our study of the effect of personality on respondents’ likelihood to complain about healthcare, we also analyzed interactions between personality and the communication illustrated in scenarios (different levels of information provision and patient involvement). Interactions are visualized in Fig. 2. Separate analyses including respondents’ satisfaction reflected the same pattern of interactions (figure not shown).

According to Fig. 2, respondents with different personality characteristics seem to perceive the physician–patient communication illustrated in scenarios somewhat differently. For example, patient involvement through SDM seems particularly appreciated in individuals with high conscientiousness scores reflected in a slightly lower wish to complain**.** Differences are small and while the effects of personality on respondents’ wish to complain are statistically significant, these effects are not statistically significantly influenced by changing the degree of patient involvement. When adjusting for personality, however, with greater patient involvement illustrated in case scenarios, respondents’ satisfaction generally increased (*P* < 0.001) while the wish to complain generally decreased (*P* < 0.001).

## Discussion

In this case vignette survey in a large, representative sample of male healthcare users, we found support for our hypothesis that satisfaction with healthcare and the wish to complain are both associated with personality factors and can be modulated through greater involvement in healthcare decision-making.

In multivariate analyses, we found higher scores on the agreeableness dimension were associated with higher patient satisfaction and lower wish to complain while high neuroticism scores were associated with lower patient satisfaction and an increased wish to complain about healthcare. In spite of personality factors, satisfaction increased with greater patient involvement illustrated in case scenarios while the wish to complain decreased. Below, we discuss the findings in detail with reference to the existing research literature.

### Discussion of study findings

An increasing number of studies have demonstrated that some patient socio-demographic characteristics may predict a higher risk of complaints [4, 6–11]. Besides, the idea that encounters with some groups of patients, including those with ‘mistrustful behavior’, can be particularly challenging appears in the literature [12, 25–28]. The evidence supporting a link between a patient’s personality and the proclivity for being dissatisfied with care (and to complain) has, however, been scant. Some research has suggested an impact of personality on patients’ satisfaction. Using the Eysenck Personality Inventory, Finlay et al. demonstrated that oral surgery patients who were dissatisfied tended to have higher neuroticism scores [29]. Using the Neuroticism-Extraversion-Openness Five-Factor Personality Inventory, Serber et al., found that higher scores on agreeableness were associated with greater patient satisfaction [13]. Similarly, using the Five-Factor Personality Inventory, Hendriks et al. found that agreeableness significantly predicted patient satisfaction [14]. In a study in patients with localized prostate cancer, Block et al. found a negative correlation between openness scores and satisfaction and a positive correlation between conscientiousness scores and satisfaction [15]. Furthermore, in a newer study of patients' satisfaction with the visual function following cataract surgery, Rudalevicius et al. found patients with neuroticism as their dominant personality trait were least happy with postoperative outcomes while those whose dominant personality traits were high conscientiousness and agreeableness demonstrated the highest satisfaction [30].

In regard to the association between health care user’s personality and their wish to complain, evidence is anecdotal at best. Nonetheless, complainants are now and then described as ‘psychologically ill’ or having ‘problematic’ personalities [16, 17]. Rowlands suggested a link between paranoid type mental disorders and malpractice litigation [31], a finding that later received some support from Allaz et al.’s study proposing a relationship between paranoid personality disorder (PPD) and litigation more generally [12]. In this regard, among five-factor model (FFM) dimensions, high neuroticism and low agreeableness appear to be most important for the conceptualization of PPD [32]. Similarly, Fishbain et al. (2007) previously found mistrust to be associated with thoughts of suing a physician [33].

Our study, using hypothetical case vignettes, supports previous findings of associations between high FFM dimensional scores in neuroticism scores and low scores in agreeableness and respondents’ complaint behavior and suggest that a corresponding reverse relationship may exist with their expressed satisfaction with care. Several explanations may account for these findings. However, one explanation may be that men low in agreeableness generally are those most likely to complain as they may tend to blame others for their own bad outcomes (negative views of others and low trust) and wish to retaliate (aggression). In parallel, while patient personality may predict satisfaction with health care and the likelihood of a complaint, it is well established that communication plays an important role in malpractice litigation. A study by Beckman et al. suggested that the decision to initiate malpractice litigation is often associated with poor delivery of information and lack of patient-physician collaboration [34] and Levinson and colleagues identified significant differences in communication behaviors of no-claims and claims physicians [35]. Later studies have supported the central role of poor communication in complaints about health care [4, 18, 36]. Hawkins and Paterson in their medico-legal audit of 100 cases found that contributory causes were failure of communication in 27 and matters connected with the patient's attitude or personality in 20 [26]. Our findings suggest that communication and patient involvement may be perceived differently by health care users with different personalities. While patient involvement in decision-making seems to be generally desired by healthcare users [37], its realization in practice may not be uniformly acknowledged across various personality styles emphasizing the need for opportunities to make individual adjustments in every patient encounter.

### Limitations

This research has several limitations. First, due to the focus on prostate cancer, our study only included men. The association between gender and complaint behavior is complex and uncertain. Women tend to report higher agreeableness and extraversion than men, [38], which could predict a lower wish to complain. On the other hand, women also tend to report higher levels of neuroticism than men [38], which could predict an increased wish to complain. While previous studies have shown that most complaints are filed by women [4, 7, 11] this effect may be mediated by women being more likely than men to be recipients of healthcare during childbearing years and to have career responsibilities for children and elders who are receiving healthcare.

Second, the case vignette design reflects hypothetical judgments and we therefore cannot be certain that participants’ behavior might have been different in real life. This caveat, however, seems contradicted in studies comparing actual choices with stated preferences [39]. Likewise, during survey development, patient and public representatives indicated that they could identify with the patient in the situations described in vignettes [22] and a large majority of survey respondents indicated that they were able to identify with the situations described in vignettes [18]. In addition, we studied participating healthcare users’ wish to complain about healthcare, rather than actual complaint behavior. Research has suggested that real life complaint figures may not necessarily reflect healthcare users’ wish to complain about healthcare [40]. Hence, conclusions from this study about the personality characteristics of patients who succeed in filing a formal complaint should be drawn with caution. Furthermore, in our analyses of the interaction of communication with personality, we only studied the effect of different levels of patient involvement. This is a simplification of the multisided aspects of provider-patient communication which include also, for example, health care provider’s personality and ability to demonstrate empathy [41]. As a final point, the possibility of non-response bias must be kept in mind. Even if our comparisons with national statistics information and previously published international data suggest our sample to be reasonably representative of the socio-demographic, personality, and decision control preferences of adult men, the possibility of residual nonresponse bias cannot be ruled out [20].

## Conclusion

While a few previous studies suggest that personality may influence satisfaction with healthcare, this study is the first to show an association between health care users’ personality characteristics and their wish to complain. Together with our finding of an overall beneficial effect of greater communication on satisfaction with healthcare, the finding that lower agreeableness and higher neuroticism are associated with lower satisfaction and increased wish to complain points to a need to better understand the concerns and communication needs of these patients.

## Supplementary Information

**Additional file 1.**

**Additional file 2.**

## Data Availability

The dataset used during the current study is available from the corresponding author on reasonable request.
