# Supplementary material for: Personality characteristics associated with satisfaction with healthcare and the wish to complain
Source: BMC Health Serv Res. 2022 Nov 1;22:1305. doi: 10.1186/s12913-022-08688-7 (PMC9628068; doi:10.1186/s12913-022-08688-7)
Supplement: Supplementary file 2 — Additional file 2. [file 12913_2022_8688_MOESM2_ESM.docx]

**Supplementary table**

**Associations between BFI dimensional scores and satisfaction resp. complaint likelihood**

| **Outcome** | **BFI dimension score** | **Crude univariate** | | **Adjusted univariate** | | **Multivariate** | |
| --- | --- | --- | --- | --- | --- | --- | --- |
|  |  | **Coef. (95% CI)** | **P-value** | **Coef. (95% CI)** | **P-value** | **Coef. (95% CI)** | **P-value** |
| Satisfaction with health care described in vignette | Extraversion | 0.02 (0.00; 0.03) | 0.008 | 0.02 (0.01; 0.03) | 0.004 | 0.00 (-0.01; 0.01) | 0.857 |
|  | Agreeableness | 0.06 (0.05; 0.08) | <0.001 | 0.06 (0.05; 0.08) | <0.001 | 0.06 (0.04; 0.07) | <0.001 |
|  | Conscientiousness | 0.02 (0.01; 0.04) | 0.002 | 0.02 (0.01; 0.04) | 0.001 | 0.01 (-0.00; 0.03) | 0.121 |
|  | Neuroticism | -0.03 (-0.04; -0.02) | <0.001 | -0.03 (-0.04; -0.02) | <0.001 | -0.02 (-0.03; -0.00) | 0.012 |
|  | Openness | -0.00 (-0.02; 0.01) | 0.418 | -0.00 (-0.02; 0.01) | 0.419 | -0.00 (-0.01; 0.01) | 0.721 |
| Complaint likelihood *) | Extraversion | -0.01 (-0.02; 0.00) | 0.093 | -0.01 (-0.02; 0.00) | 0.130 | 0.01 (-0.00; 0.03) | 0.051 |
|  | Agreeableness | -0.08 (-0.09; -0.06) | <0.001 | -0.07 (-0.09; -0.06) | <0.001 | -0.07 (-0.08; -0.05) | <0.001 |
|  | Conscientiousness | -0.03 (-0.04; -0.01) | <0.001 | -0.02 (-0.04; -0.01) | 0.001 | -0.01 (-0.03; 0.00) | 0.093 |
|  | Neuroticism | 0.05 (0.04; 0.07) | <0.001 | 0.04 (0.03; 0.05) | <0.001 | 0.04 (0.03; 0.06) | <0.001 |
|  | Openness | -0.01 (-0.02; 0.01) | 0.400 | 0.01 (-0.00; 0.02) | 0.241 | -0.01 (-0.02; 0.00) | 0.096 |
| Likelihood of disciplinary complaint | Extraversion | -0.01 (-0.03; -0.00) | 0.038 | -0.01 (-0.02; -0.00) | 0.049 | 0.01 (-0.00; 0.02) | 0.167 |
|  | Agreeableness | -0.08 (-0.09; -0.06) | <0.001 | -0.07 (-0.09; -0.06) | <0.001 | -0.07 (-0.08; -0.05) | <0.001 |
|  | Conscientiousness | -0.03 (-0.04; -0.01) | 0.001 | -0.03 (-0.04; -0.01) | 0.001 | -0.01 (-0.03; 0.00) | 0.081 |
|  | Neuroticism | 0.05 (0.03; 0.06) | <0.001 | 0.04 (0.03; 0.05) | <0.001 | 0.04 (0.02; 0.05) | <0.001 |
|  | Openness | -0.01 (-0.02; 0.00) | 0.221 | 0.00 (-0.01; 0.02) | 0.595 | -0.01 (-0.03; 0.00) | 0.050 |
| Likelihood of compensation claim | Extraversion | -0.01 (-0.02; 0.01) | 0.263 | -0.01 (-0.02; 0.01) | 0.361 | 0.02 (0.00; 0.03) | 0.027 |
|  | Agreeableness | -0.08 (-0.10; -0.06) | <0.001 | -0.07 (-0.09; -0.05) | <0.001 | -0.07 (-0.09; -0.05) | <0.001 |
|  | Conscientiousness | -0.03 (-0.04; -0.01) | 0.003 | -0.02 (-0.04; -0.01) | 0.009 | -0.01 (-0.03; 0.01) | 0.174 |
|  | Neuroticism | 0.05 (0.04; 0.07) | <0.001 | 0.04 (0.03; 0.06) | <0.001 | 0.05 (0.03; 0.06) | <0.001 |
|  | Openness | -0.00 (-0.02; 0.01) | 0.731 | 0.01 (-0.00; 0.02) | 0.104 | -0.01 (-0.02; 0.01) | 0.255 |

*) Combined likelihood of compensation claim and disciplinary complaint
